# Mycophagy in Primates of the Issa Valley, Tanzania

**DOI:** 10.1002/ece3.72000

**Published:** 2025-09-25

**Authors:** Theresa A. Schulze, Wiske Bovee, Jacqueline Loos, Jane Lukumay, Vicky M. Oelze, Noah Siegel, Fiona A. Stewart, Alex K. Piel

**Affiliations:** ^1^ Leipzig University Leipzig Germany; ^2^ Van Hall Larenstein Leeuwarden the Netherlands; ^3^ University of Vienna Vienna Austria; ^4^ GMERC, Ltd. Mpanda Tanzania; ^5^ Department of Anthropology University of California Santa Cruz California USA; ^6^ Northeast Mycological Federation Royalston Massachusetts USA; ^7^ Department of Anthropology University College London London UK; ^8^ Department of Human Origins Max Plank Institute for Evolutionary Anthropology Leipzig Germany

**Keywords:** African primates, chimpanzees, diet, fungivory, miombo woodland, mushroom

## Abstract

Mycophagy (fungi consumption) is an important animal‐ecosystem interaction and provides nutrients for numerous mammalian taxa, such as primates. Although mushroom consumption is a widespread behavior documented in about a quarter of all known primate species, surprisingly little is known about their use of mushrooms as either a staple or fallback food and the relationship between mushroom availability and consumption. We used direct observational data on mushroom consumption in the diet of three sympatric primate species: chimpanzees ( *Pan troglodytes schweinfurthii* ) and red‐tailed monkeys ( *Cercopithecus ascanius* ) from 2019 to 2022, and yellow baboons ( *Papio cynocephalus* ) from 2015 to 2022 in a mosaic woodland habitat in the Issa valley, western Tanzania, to address these gaps. We analyzed mushroom consumption patterns and assessed mushroom availability from line transects for a period of 15 months (Oct 2022—Dec 2023). Our findings show that mushrooms were a consistent dietary component for all three species during the wet season (October–April; chimpanzees—4%, baboons—17% and red‐tailed monkeys—4%), while baboons also continued to consume mushrooms (~11% of their annual diet) even when availability was low outside the wet season. This is also the first report of mushroom consumption by red‐tailed monkeys. We conclude that mushrooms serve as a fallback resource for Issa chimpanzees and red‐tailed monkeys, while they represent a preferred food for Issa baboons. We contextualize mushroom consumption as a potential strategy of niche partitioning to reduce interspecific feeding competition and underscore the importance of mycophagy and its role in primate dietary ecology and human evolution.

## Graphical abstract

Mushroom consumption in Issa Valley primates varies by species and availability. Chimpanzees and red‐tailed monkeys use mushrooms as a fallback food, while baboons prefer them year‐round. Our study highlights the ecological and evolutionary significance of mycophagy in primates.

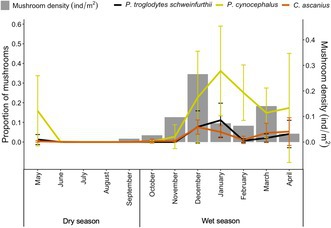

## 1 Introduction

Fungi are an important, globally recognized food for humans and wildlife alike (Li et al. 2021). They deliver nutritional value for people wherever they are consumed and, in many cases, have cultural and economic value also (Rizzo et al. 2021; Roncero‐Ramos and Delgado‐Andrade 2017). Fungi are a component of the dietary repertoire of over 500 mammal species across 15 Orders (Elliott et al. 2022). For instance, Diprotodontia, a group of marsupial mammals native to Australia, New Guinea and the surrounding islands, comprise various species that predominantly consume fungi (Elliott and Vernes 2021; Nuske et al. 2017, 2019; Vernes and Lebel 2011). Further, there are several orders across placental mammals that consume fungi, including Artiodactyla, Carnivora and Rodentia, all of which are also dispersers of fungal spores (Baskin and Danell 2003; Castelló 2016; Elliott, Georgiev, et al. 2020; Elliott, Townley, et al. 2020; Schwartz and Schwartz 2001; reviewed in Elliott et al. 2022).

Non‐human primates (primates hereafter) are well known seed dispersers (Chapman 1995), with gut passage times ranging from ~14 to 85 h (reviewed in Lambert 2002). Over that period of time, daily path distances vary with species, with smaller species ranging < 500 m and larger up to 4 km on average (e.g., for red‐tailed monkeys see McLester et al. 2019 and for chimpanzees see Green et al. 2020). They also consume a diversity of fungi (Elliott et al. 2022; Hanson et al. 2003; Sawada et al. 2014). Fungi typically comprise < 5% of their diet (Hanson et al. 2003) and although there has been extensive research into mushroom nutrition for humans and wildlife, compared to non‐primates, there are relatively few reports investigating the role of fungi for primates (Elliott et al. 2022). Moreover, while reports have described climatic triggers for fruiting events (Boddy et al. 2014; Boukary et al. 2024), these patterns have never been described in analyses of resource availability for primates.

Of the ~500 recognised primate species, mycophagy (the consumption of fungi) has been described in 105 species from 13 families (Elliott et al. 2022). Despite the diverse taxonomic representation of primate mycophagy, fungi consumption does not typically represent a significant proportion of primate feeding time (Hanson et al. 2003). In most descriptions of fungi consumption by primates, fungi species are rarely identified, further limiting our broader understanding of fungi diversity in primate diet.

Exceptions do exist, however. Hanson et al. (2006) described the nutritional composition of four fungal species (*Ascopolyporus polyporoides*, *As. polychrous*, *Auricularia auricula* and *Au. delicata*) consumed by Goeldii's monkey (*Callimico goeldi*) in Bolivia. The authors argued that the consumption of these fungi played a role in differentiating diet between Callitrichines, with the Goeldii's monkey relying on fungi rather than fruit during times of fruit scarcity, compared to the brown‐mantled tamarin ( *Saguinus fuscicollis* ) and the white‐lipped tamarin ( *Saguinus labiatus* ), which preferred fruit. In Bornean red langurs (*Presbyits rubicunda*), fungi consumption was analyzed from direct observations and camera traps and represented only 1% of feeding observations (Cheyne et al. 2019). The authors described the consumption of seven different fungi species, from four different families: Boletaceae, Entolomataceae, Russulaceae and Polyporaceae. None of these types were consumed by local people.

Only two other studies describe whether fungi serve as staple or fallback foods in primates. In bonobos ( *Pan paniscus* ) from Lui Kotale, Dem. Rep. Congo and Japanese macaques ( *Macaca fuscata yakui* ), fungi consumption increased with increasing fruit abundance, indicating both species supplement nutrients through mycophagy, but that such foods were not fallback foods (Lucchesi et al. 2021; Sawada et al. 2014). Further, lowland gorillas forage on truffles opportunistically, consuming fungi throughout the year. Still, there is marked variation in consumption frequency between social groups, suggesting that fungi function more as complementary or occasional foods rather than staple or fallback resources (Abea et al. 2024). However, neither study reported on temporal patterns of fungi availability. That is, whereas studies abound on fruit, leaf and invertebrate seasonal abundance changes for questions on primate dietary ecology (Potts et al. 2011; van Schaik and Noordwijk 1985), we could find no study that reported on fungi availability concurrent to the study of primate mushroom consumption, making it nearly impossible to draw inferences on whether fungi are a preferred food source or not, and how consumption changes with, for example, fruit availability.

To address this knowledge gap, we investigated mushroom consumption across three sympatric primate species—chimpanzees ( *Pan troglodytes* ), yellow baboons ( *Papio cynocephalus* ) and red‐tailed monkeys ( *Cercopithecus ascanius* ) living in a mosaic habitat in the Issa valley, western Tanzania. Our study focused specifically on mushrooms, macrofungi with visible fruiting bodies; we did not consider truffles, truffle‐like and other hypogeous fungi. At Issa, chimpanzee diet is comprised of fruit, leaves, invertebrates (termites and ants) and vertebrates (Phillips et al. 2023; Piel et al. 2017; Ramirez‐Amaya et al. 2015; Stewart and Piel 2014). To date, there has been no report of mushroom consumption in Issa chimpanzee diet, although the only previous study that described the diversity of Issa chimpanzee diet relied on macroscopic (faecal) analysis (Piel et al. 2017), which does not allow for the detection of fungal remains and therefore cannot determine whether fungi were part of the diet. In Issa's baboons, Mkola et al. (2021) described mushrooms to comprise 7.8% of their annual diet. There are no published data on red‐tailed monkey diet from the study area. Additionally, while mushroom consumption has been reported in chimpanzees and yellow baboons from other study sites, it has never been the primary focus of investigation; therefore, no quantitative data on feeding proportions are available (Altmann 2009; Kitegile 2023; Matthews et al. 2019, 2020; Post 1982; Stacey 1986).

Given the widespread consumption of mushrooms by humans throughout Tanzania (Tibuhwa 2013) and the previous reports of primate consumption (Elliott et al. 2022; Hanson et al. 2003; Sawada et al. 2014), we expected mushrooms to play a key role in the Issa primate diet. Extensive mycophagy at Issa would have multiple implications. First, given the analogous vegetation of Issa to paleoecological reconstructions of hominin habitats (Drummond‐Clarke et al. 2022), identifying the importance of mycophagy for Issa's primates would implicate this resource not only for the extant primate community there but also for hominids that may have exploited a key (mostly) terrestrial resource (McKenna 1999; Sayers and Lovejoy 2014). Further, if we find mushrooms to be an important resource (defined here as > 5% of consumed foods) across Issa's primate community, it would prompt follow‐up studies on niche partitioning and how these three consumer species compete for a seasonally restricted food source. Mushrooms might have the potential to inform the ranging patterns and habitat use of the three primate species. Finally, with mushrooms being an essential part of some local (human) diet (Tibuhwa 2013) and sold at markets throughout the country (Chelela et al. 2015) there is potential for human‐wildlife conflict over a spatiotemporally limited wild food resource.

As chimpanzees are ripe fruit specialists, we hypothesized that they would prefer fruit at all times of year and only consume mushrooms during fruit‐scarce months; whereas baboons would prefer mushrooms, especially in an effort to avoid food competition with chimpanzees (Matsumoto‐Oda and Kasagula 2000; Nishida 2002). We expected mushrooms to be a staple (consumed as available) food for red‐tailed monkeys and to comprise a modest proportion of their diet, given the monkeys' arboreal nature and the tendency for mushrooms to be ground‐dwelling (Bödeker et al. 2016).

## 2 Methods

### 2.1 Study Site

**FIGURE 1:**
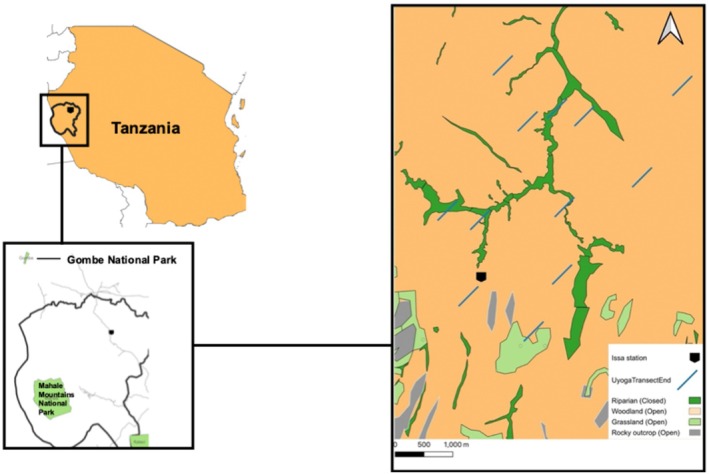
Location of Issa study area in Tanzania with vegetation types and mushroom line transect locations (blue lines).

The Issa Valley is part of the Greater Mahale Ecosystem in western Tanzania (Figure 1). The study area consists of numerous large valleys divided by mountains and plateaus, with altitude varying between 1150 and 1800 m above sea level (Drummond‐Clarke et al. 2022). The habitat is considered a mosaic woodland, dominated by deciduous miombo woodlands (*Brachystegia* and *Julbernardia* dominated), and including seasonally inundated grasslands with strips of evergreen, riparian forests. We collapsed vegetation into three vegetation types: riparian forests, miombo woodland and grasslands. These evergreen forest patches comprise only 7% of the total land area and harbor forest‐dwelling fauna such as blue duiker ( *Philantomba monticola* ) and red‐tailed monkey. Other common mammalian species found at Issa include bushbuck ( *Tragelaphus scriptus* ), common duiker (*Sylviacapra grimmia*), roan antelope ( *Hippotragus equinus* ) and klipspringer ( *Oreotragus oreotragus* ) (Piel et al. 2019), in addition to numerous other small mammal species (D'Ammando et al. 2022). Issa experiences two distinct seasons: a dry season from May to September and a wet season from October to April. The mean annual temperature during the study period was 23°C; the average rainfall for the study period was 1030 mm.

### 2.2 Study Subjects

We collected data on a single community of chimpanzees and single troops of yellow baboons and red‐tailed monkeys. Researchers searched for chimpanzees ~20 days per month, although the number of focal follows and observation time varied. Baboons and red‐tailed monkeys were followed for up to 6 consecutive days per month. The Issa chimpanzee community ranged in size from 28 to 32 animals during the study. The community was fully habituated in summer 2018, with all individuals uniquely recognisable. Adults were followed from morning nest until evening nest and followed from approximately 15 m (Giuliano et al. 2022). The ‘camp’ baboon troop was first habituated in 2011, and group size ranged from 8 to 18 individuals during the initial study period (Johnson et al. 2015). The red‐tailed monkeys were initially a single troop of more than 50 individuals but split into two troops in 2016, after which only one troop has been studied (Fornof et al. 2023). Observations of this single troop were included in the current study. Although some members of the focal troop were individually recognisable by physical features, because not all were known at the time of the study, we used age‐sex class to describe the focal monkey.

### 2.3 Mushroom Consumption

To assess mushroom consumption, we analyzed behavioral data collected from chimpanzees and red‐tailed monkeys between January 2019 and August 2023. For baboons, we used data between January 2015 and December 2022 as we started habituating a different group from January 2023 onwards. During follows of each species, we performed focal animal sampling to collect 5‐min interval data on feeding activity, focusing on feeding events. We followed one individual as long as possible on a given day. If we lost sight of the focal individual for more than 30 min, we initiated a new follow of a different individual for baboons and chimpanzees. In contrast, for red‐tailed monkeys, we switched to a new focal individual with distinct physical features from the previous one. For each feeding event, we recorded the food item type (mushroom, fruit, animal prey, leaves, flowers, bark, roots, insects, bud, others) and local or scientific name when known. When possible, whenever a focal individual was feeding on a mushroom, we photographed or collected a sample of the species consumed for later identification.

Ideally, mushroom consumption would have further been quantified via spore identification in macroanalysis of faeces (Rafferty et al. 1994) or else using flotation or molecular techniques (see Bazzle et al. 2014; Castaño et al. 2017). However, both because we did not initiate the dietary study with a focus on fungi and also lacked local expertise and equipment, we did not invoke these approaches.

### 2.4 Mushroom and Fruit Abundance

To assess and monitor the spatiotemporal patterns of mushroom availability, we established 12 × 500 m transects, placed across the study site (following Burrola‐Aguilar et al. 2013). We distributed transects across different vegetation types: miombo woodlands (*n* = 9), riparian forests (*n* = 2) and grassland (*n* = 1). We walked each transect once per month between October 2022 and December 2023. Surveys were conducted on approximately the same days each month, regardless of weather conditions. For each transect, we noted the presence of every mushroom within 2 m of each side of the transect. The total survey area included 24,000 m^2^ (6000 × 4 m). We calculated mushroom density as individuals per square meter. For each month we divided the total number of mushrooms found by the total area surveyed (e.g., in February 2023 we surveyed 12 transects (12 × 500 × 4 m) and found in total 509 mushrooms, the respective calculation was: $\frac{509}{12\times 500\times 4\;m}$). For the current study, we were not able to identify mushrooms to the taxonomic level and so collapsed them all into a single category.

To assess mushroom consumption against fruit availability, we used monthly phenology data of chimpanzees top feeding species (Piel et al. 2017), where we visited more than 900 trees once per month and recorded fruit availability using phenology scores from 0 to 4: 0, none; 1, 1%–25%; 2, 26%–50%; 3, 51%–75%; 4, 75%–100% (percentages represent the proportion that the tree could otherwise hold). We also extracted species density from 450 20 × 20 m botanical plots where each tree that was taller than 1 m and had a diameter at breast height (DBH) larger than 10 cm was identified.

### 2.5 Data Analysis

We calculated a monthly Fruit Availability Index (${\mathrm{FAI}}_{m}$) following Aristizabal et al. (2019) and using the formula:

$${\mathrm{FAI}}_{m}=\sum B_{k}\times D_{k}\times P_{\mathrm{km}}$$

where $B_{k}$ represents the total tree basal area per hectare for species k that refers to the cross‐sectional area of a tree trunk at breast height. Further, $D_{k}$ denotes the species density per hectare for species k and $P_{\mathrm{km}}$ the phenology score for species k in month m. While the calculated FAI reflects the top 20 fruit species for chimpanzees, it does not specifically represent the primary fruit species of red‐tailed monkeys and baboons. However, since all three species are considered frugivorous and dietary overlap among them has been documented elsewhere (Matsumoto‐Oda and Kasagula 2000; Tweheyo and Obua 2001), the FAI still serves as a useful proxy for general fruit availability for all consumer species.

Additionally, we calculated density estimates for all aggregated mushroom types following Buckland et al. (2010) for each transect. We then performed Spearman Rank correlations to determine whether FAI or mushroom density is associated with rainfall.

For each species, we then calculated the average proportion of mushroom feeding events for each month by dividing the number of mushroom feeding events per month by the total number of feeding events of that month, across the study period. Further, to account for inter‐monthly variation, we conducted a Kruskal–Wallis Test in R (version 4.4.3) to compare consumption patterns among the species; subsequently, Dunn's post hoc tests were conducted to identify inter‐group variation. In addition, we ran a Spearman correlation for each species to assess whether the proportion of mushrooms consumed was associated with FAI (to test hypotheses about the role of mushrooms as fallback foods).

To investigate the potential relationship that ranging patterns and habitat use may have with mushroom presence and density, we stratified the calculated density by vegetation type (riparian forest, miombo woodland and grassland) for all transects with associated vegetation type recorded (these data were absent for September to December 2023). We then compared density across vegetation types using a Kruskal–Wallis test followed by Dunn's post hoc tests.

## 3 Results

**FIGURE 2:**
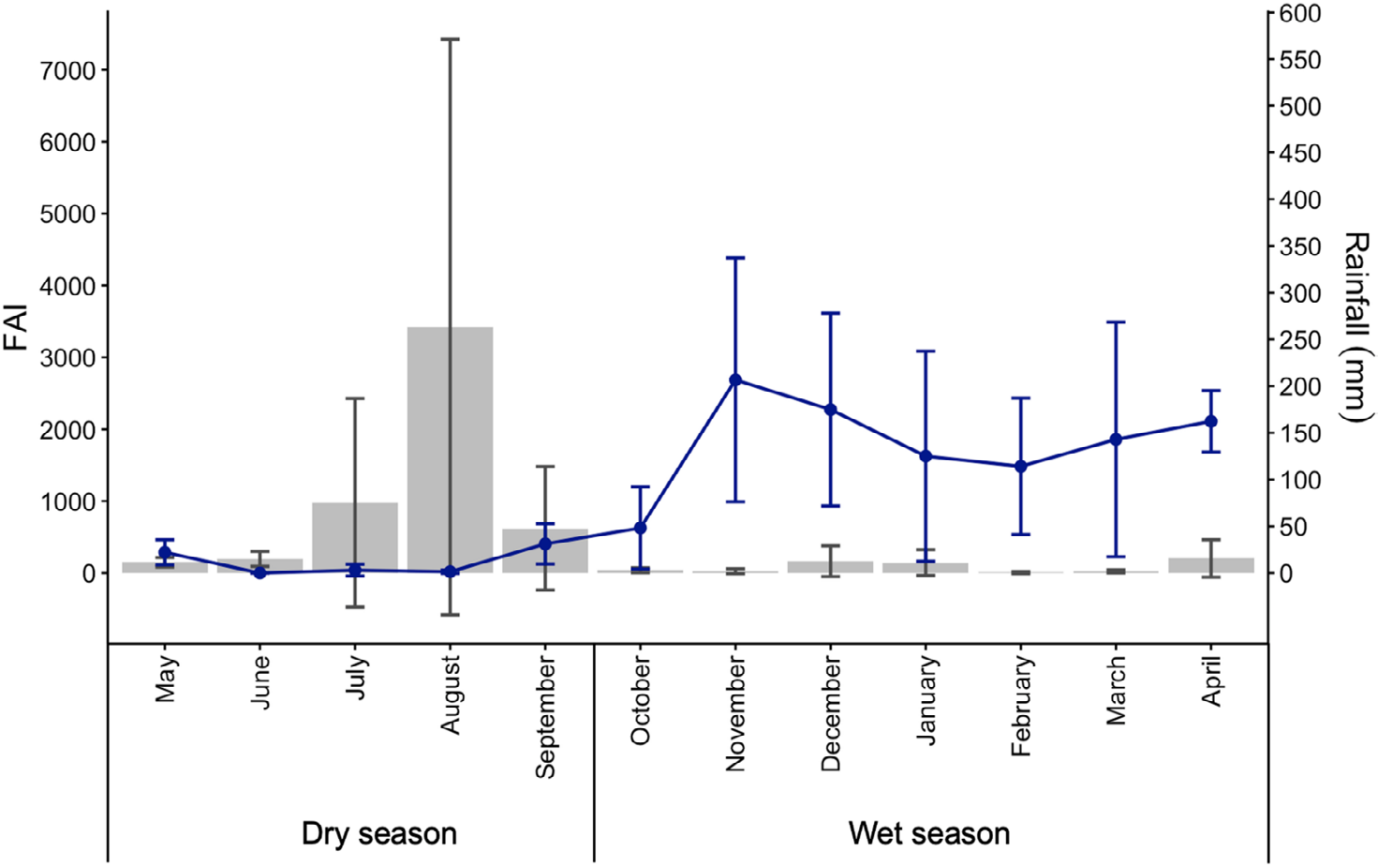
Monthly average in rainfall (blue line, vertical lines represent 0.25 and 0.75 quantiles) and fruit availability index (FAI, grey bars, vertical lines represent 0.25 and 0.75 quantiles). FAI peaks in July–August, whereas the onset of the rains typically occurs in late September to October.

Between 2019 and 2023, we compiled a dataset of 23,878 feeding events of chimpanzees (numbers of months of observation: 54; average feeding events per month: 442.19 ± 404.04), 10,440 feeding events of baboons (numbers of months of observation: 74; average feeding events per month: 141.08 ± 130.92) and 18,064 feeding events of red‐tailed monkeys (numbers of months of observation: 53; average feeding events per month: 340.83 ± 253.82). Between 2022 and 2023, we walked 69 km of line transects and observed 6361 individual mushrooms in that period. We found a temporal offset between FAI (Fruit Availability Index) and the onset of the rains (*ρ* = −0.42, *p* = 0.002), with FAI peaking in July–August each year, and the rains beginning about 1–2 months later (Figure 2). Mushroom density was positively correlated with rainfall (*ρ* = 0.777, *p* < 0.001).

Overall, we found a significant difference in monthly mushroom consumption between the three primate species (*χ* ^2^ (2) = 10.681, *p* = 0.004). Chimpanzee consumption differed significantly from baboons (*z* = 3.26, *p* < 0.001). Additionally, we found a tendency towards a difference in mushroom consumption patterns between chimpanzees and red‐tailed monkeys (*z* = −1.52, *p* = 0.064), as well as between baboons and red‐tailed monkeys (*z* = 1.60, *p* = 0.055). Across the entire observation period, approximately 11% (SD = 12.48%) of baboon feeding observations were on mushrooms, compared to 2% (chimpanzees: SD = 3.63%, red‐tailed monkeys: SD = 2.74%) for both chimpanzees and red‐tailed monkeys, respectively (Table 1).

**TABLE 1:** Dietary proportions of major food types for each primate species.

	Chimpanzees	Baboons	Red‐tailed monkeys
Fruit	0.53	0.19	0.28
Flower	0.11	0.03	0.19
Leaves	0.12	0.05	0.2
Invertebrates	0.06	0.10	0.29
Seeds/pods	0.14	0.26	0.01
Grass		0.05	
Roots		0.18	
Mushrooms	0.02	0.11	0.02

**FIGURE 3:**
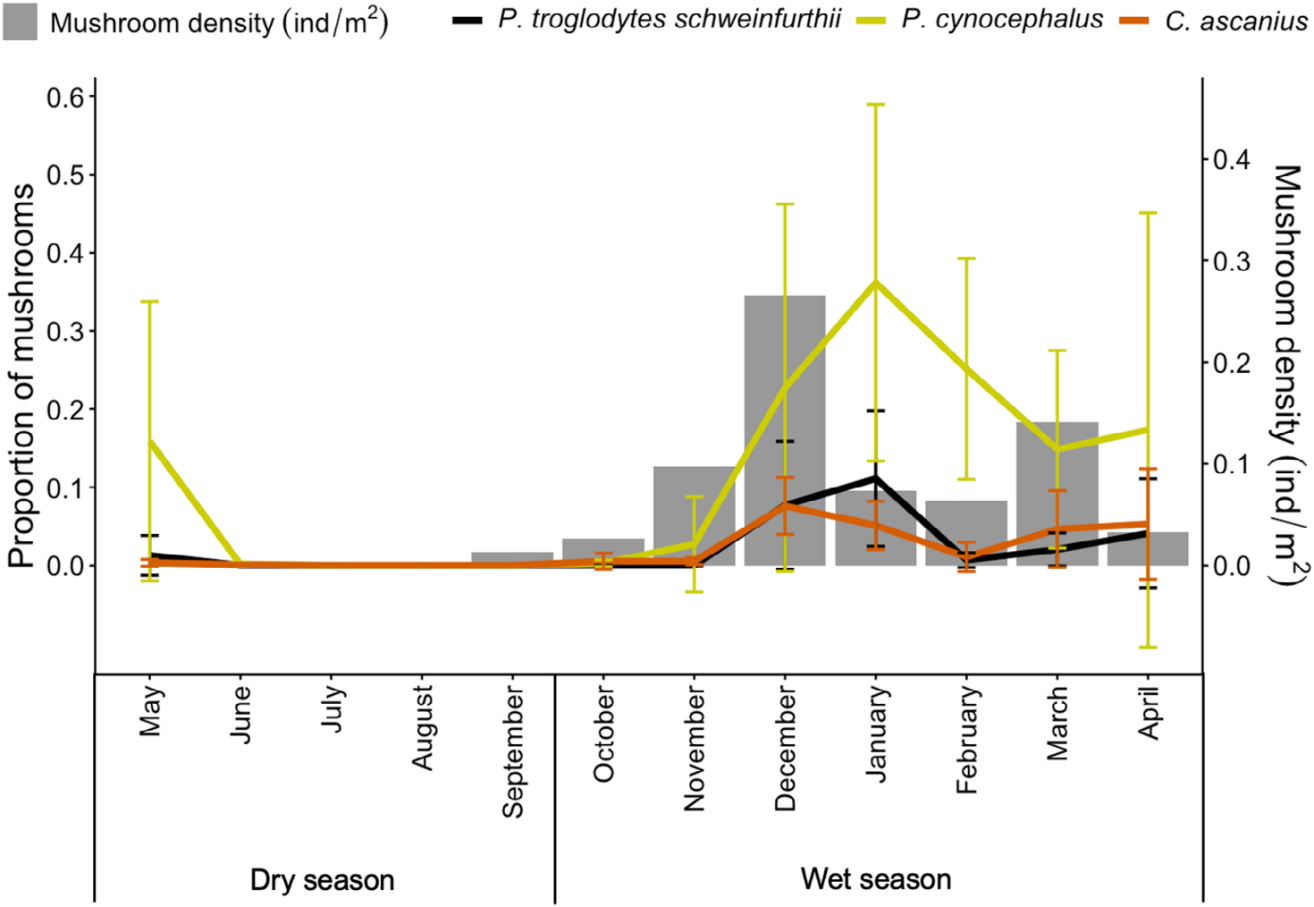
Monthly mushroom consumption for each primate species (chimpanzees: black; yellow baboons: yellow; red‐tailed monkeys: red; vertical lines represent 0.25 and 0.75 quantiles) as compared to mushroom density (ind/m^2^; grey).

When we looked at monthly mushroom consumption, the inter‐specific differences became even more striking, with peaks of mushroom consumption in chimpanzees (11%) and baboons (36%) in January, and highest consumption rates in red‐tailed monkeys (8%) in December (Figure 3). Whilst there were recorded instances of primates eating mushrooms in both May and June (baboons—8%, chimpanzees and red‐tailed monkeys < 1%), we detected no mushrooms on transects during most of the dry season, with none observed between May and August, and only 52 in September. However, whereas chimpanzees (4%) and red‐tailed monkeys (4%) exhibited moderate consumption during the mushroom‐abundant (wet) season (October—April), baboons relied on mushrooms far more over that same period (17%; Figure 3). Overall, mushrooms were a key dietary component for chimpanzees from December to January, for red‐tailed monkeys in December, January and April, and for baboons from December to May. Peak (monthly) consumption varied dramatically from 7.6% (red‐tailed monkeys), 11.1% (chimpanzees), to 36.6% (baboons; Figure 3). Chimpanzees and red‐tailed monkeys' consumption roughly tracked mushroom abundance, whereas baboons continued to feed on mushrooms even when mushroom availability was low.

Further, there was no correlation between chimpanzee mushroom consumption and fruit availability (*ρ* = −0.13, *p* = 0.344); while baboons and red‐tailed monkeys exhibited a tendency towards increased mushroom consumption when fruit availability declined (baboon: *ρ* = −0.28, *p* = 0.064; red‐tailed monkey: *ρ* = −0.26, *p* = 0.063).

When we compared mushroom density across the three different vegetation types to investigate its potential impact on ranging patterns and habitat use, we found significant differences (*χ* ^2^ (2) = 9.65, *p* = 0.008). Mushrooms were most abundant in woodlands (7.002 ind/m^2^) then forests (2.786 ind/m^2^) and lastly, grasslands (0.004 ind/m^2^). The maximum mean density for riparian forests occurred in November at 0.646 ind/m^2^ (SD = 0.673 ind/m^2^), while the maximum mean density for miombo woodland was recorded in December at 0.339 ind/m^2^ (SD = 0.171 ind/m^2^). Mushrooms were rare in grasslands, with a peak of only 0.004 ind/m^2^ in January.

## 4 Discussion

This study contributes to better understanding the role of mushrooms as food sources for a primate community, with implications for niche partitioning. Our study revealed that mushrooms formed > 5% of the diet for three sympatric primate species for some months during the wet season. Dietary importance can be assessed in the context of dietary diversity. For example, of the 77 plant species consumed by Issa chimpanzees, many of which are often only available for 1 to 2 months of the year, the top twelve most consumed species each represent more than 5% of their diet (Piel et al. 2017). Baboons consumed mushrooms as a large proportion of their diet even when mushroom availability was low, suggesting that baboons seek them out regardless of their relative abundance.

Red‐tailed monkeys exhibited mushroom consumption patterns that tracked mushroom availability, for example, as monthly mushroom density rose, so did consumption proportions. Both baboons and chimpanzees reached their maximum mushroom consumption 1 month following peaks in mushroom density. For baboons, however, their consistent level of consumption, both when mushroom density and chimpanzee consumption fell, suggests that mushrooms are a preferred food (Lambert and Rothman 2015), and one that may mitigate inter‐specific feeding competition.

Chimpanzees and baboons are sympatric in numerous areas across their distribution, but there are few studies examining feeding competition. At Mahale, in the only direct examination of feeding competition, overlap between chimpanzee and baboon foods reached 50% for ripe fruits, but the authors suggested that the far broader dietary repertoire for baboons likely mitigated feeding competition (Matsumoto‐Oda and Kasagula 2000; Nishida 2002). There was no mention of either (primate) species consuming mushrooms or any other kind of fungi. At Ngogo, in Kibale National Park (Uganda), chimpanzees and olive baboons ( *P. anubis* ) consumed a similar proportion of fibrous foods each month, and drivers of consumption were attributed to fruit availability, rather than inter‐specific competition (Wrangham et al. 1991). Later analyses by the same authors comparing chimpanzees and sympatric cercopithecines (including red‐tailed monkeys) showed monkeys to consume lower quality diets with higher antifeedant compounds. Here, the authors argued that monkeys avoided competition through niche partitioning (termed ‘dietary segregation’ in the study) (Wrangham et al. 1998), which may explain Issa baboon mushroom consumption as well.

At Issa, without knowing which mushroom species are being consumed, we can only speculate on whether there is direct, inter‐specific feeding competition. That is, there may still be niche partitioning even during temporally simultaneous mushroom consumption if, for example, chimpanzees and monkeys prefer different mushroom types. However, on the assumption that mushrooms hold similar benefits (e.g., nutrient composition) or dangers (e.g., toxins) for each consumer species, we suggest a scramble for mushrooms when they are available. Subsequent studies will need to identify which mushroom species are being targeted and avoided, and which nutritional properties mushrooms provide for the different consumer species.

Indirectly, we can look for niche partitioning via vegetation type. Mushroom density was highest in riparian forests in November and in miombo woodlands in December. Since chimpanzees' consumption of mushrooms peaks in January, it is likely that they prefer mushrooms that grow in woodland vegetation. Red‐tailed monkeys, which are primarily forest foragers (Sarmiento et al. 2001), exhibited a peak in mushroom consumption in December, aligned with the forest mushroom peak, while baboons foraged mainly in miombo woodlands (Norton et al. 1987; Post 1982) with a peak in mushroom consumption in January and February. Chimpanzees are generally thought to be preferential forest foragers like red‐tailed monkeys (Goodall 1986; Wrangham 1977), although miombo woodlands serve as an important foraging habitat (Piel et al. 2017). Chimpanzees exploit food in woodlands and forests at Issa (Giuliano et al. 2022) and thus may compete for mushrooms with baboons in woodlands and red‐tailed monkeys in forests. This likely overlap suggests niche partitioning at Issa; however, further examination of how mushroom consumption patterns interact with ranging is needed.

### 4.1 Human‐Primate Competition

Fungi are not only important for nonhuman primates; they serve as nutritional, economic and cultural resources for indigenous people across the world. The role of fungi across ethnic groups varies, however. For example, in northern Tanzania, the Maasai are considered mycophobic compared to the nearby and mycophilic, Kurya (Tibuhwa 2012). As with other groups, the Kurya ethnomycological knowledge is in danger of being lost, as younger people spend increasingly less time harvesting fungi with their elders (Tibuhwa 2012). In central Tanzania, work has centered more broadly on indigenous use of forest foods with vastly more modest use of fungi (e.g., three fungi species consumed over 180 households surveyed across 900 km in the Eastern Arc Mountains) (Msuya et al. 2010). Further studies are necessary to investigate the importance of fungi for humans in the study region. Once the mushrooms consumed by Issa's primates are fully identified, we can then compare consumed species by both humans and primates. Such comparisons could help to detect and better understand interspecific resource competition as well as how regional conservation strategies could incorporate an ethnoprimatological framework into their mission.

### 4.2 Limitations

This study has several limitations. First, the study lacked specific FAI for baboons and red‐tailed monkeys. For comparisons between mushroom consumption and FAI, we used the calculated FAI for chimpanzees and applied this to each (consumer) species. All three species are considered frugivorous and exhibit some dietary overlap (chimpanzees—baboons: Matsumoto‐Oda and Kasagula 2000; chimpanzees—red‐tailed monkeys: Tweheyo and Obua 2001); however, future analyses should include species‐specific FAI for each species given dietary variability (Wrangham et al. 1998). Second, we were unable to reliably or consistently identify individual consumers or individual mushrooms to taxonomic level during the study. To address this gap, we have included in Table 2 some identifications of consumed mushroom species made by a mycologist (NS) from direct and video observations of primates consuming mushrooms. For subsequent studies, we hope to use molecular techniques to help us identify mushroom types to genus if not species level (following Bazzle et al. 2014; Castaño et al. 2017). Third, observation sample sizes differed between consumer species and fluctuated across months, which may have influenced how we interpret the differences in dietary proportions. This variability may have also been affected by individual‐level variation, for which we could not account (following other similar studies, e.g., Overdorff et al. 1997). Finally, the extent to which primates function as dispersers cannot be assessed without testing primate faeces for the presence of spores and spore viability in relation to their consumption of fungi.

**TABLE 2:** Mushroom species consumed by baboons, chimpanzees, and humans from the local area (N. Siegel and J. Lukumay, pers. obs.). No data on mushroom consumption by red‐tailed monkeys were available.

	Baboons	Chimpanzees	Human
*Cantharellus platyphyllus*		x	x
*Cantharellus symoensii*	x		x
*Lactifluus rubiginosus*	x		x
*Lactifluus* cf. *heimii*	x		
*Lactifluus* cf. *pelliculatus*	x	x	
*Lactifluus edulis*	x	x	x
*Lactifluus* sp. ‘EDC 12‐068’	x		
*Lactifluus* sp.		x	
*Russula cellulata*	x		
*Russula* cf. *phaeocephala*	x		
*Russula* sp.	x		
*Termitomyces* cf. *aurantiacus*		x	x
*Termitomyces* cf. *titanicus*		x	x

## 5 Conclusion

This study provides insights into the consumption patterns of mushrooms among three sympatric primate species in the Issa Valley. Mushroom consumption was temporally and proportionally variable, with baboons preferring mushrooms as staple food and consuming them consistently even when availability was low. In comparison, chimpanzees and red‐tailed monkeys consumed mushrooms primarily as fallback foods. While red‐tailed monkeys exhibited consumption patterns that followed mushroom density, chimpanzees displayed delayed consumption peaks. This pattern suggests that mushrooms serve as a key resource for primates, and the observed differing ecological foraging strategies may indicate a mitigation of interspecific competition for food and habitat. The variation in mushroom consumption could indicate potential niche partitioning among these species, especially as red‐tailed monkeys forage in forests, while baboons and chimpanzees are mostly savanna dwelling and may forage mostly in miombo woodlands. We recommend future research to prioritize the identification of mushroom species consumed and their nutritional value to deepen our understanding of the relationship between primates and fungi.

## Author Contributions

**Theresa A. Schulze:** conceptualization (equal), data curation (lead), formal analysis (lead), investigation (equal), methodology (equal), project administration (equal), resources (equal), software (lead), validation (lead), visualization (lead), writing – original draft (equal), writing – review and editing (lead). **Wiske Bovee:** investigation (supporting), methodology (supporting), writing – review and editing (equal). **Jacqueline Loos:** funding acquisition (supporting), investigation (supporting), supervision (supporting), writing – review and editing (equal). **Jane Lukumay:** writing – review and editing (equal). **Vicky M. Oelze:** writing – review and editing (equal). **Noah Siegel:** methodology (equal), writing – review and editing (equal). **Fiona A. Stewart:** conceptualization (equal), funding acquisition (equal), investigation (equal), project administration (equal), supervision (supporting), writing – review and editing (equal). **Alex K. Piel:** conceptualization (equal), data curation (supporting), formal analysis (supporting), funding acquisition (equal), investigation (equal), methodology (equal), project administration (equal), resources (equal), supervision (lead), validation (supporting), visualization (supporting), writing – original draft (equal), writing – review and editing (equal).

## Conflicts of Interest

The authors declare no conflicts of interest.

## Data Availability

The data that support the findings of this study are openly available in Dryad at https://doi.org/10.5061/dryad.d2547d8cj.
